# Remote Sampling with Applications to General Entanglement Simulation

**DOI:** 10.3390/e21010092

**Published:** 2019-01-19

**Authors:** Gilles Brassard, Luc Devroye, Claude Gravel

**Affiliations:** 1 Département d’Informatique et de Recherche Opérationnelle, Université de Montréal, Montréal, QC H3C 3J7, Canada; 2 Canadian Institute for Advanced Research, Toronto, ON M5G 1M1, Canada; 3 School of Computer Science, McGill University, Montréal, QC H3A 0E9, Canada; 4 National Institute of Informatics, 2-1-2 Hitotsubashi, Chiyoda, Tokyo 101-0003, Japan

**Keywords:** communication complexity, quantum theory, classical simulation of entanglement, exact sampling, random bit model, entropy

## Abstract

We show how to sample exactly discrete probability distributions whose defining parameters are distributed among remote parties. For this purpose, von Neumann’s rejection algorithm is turned into a distributed sampling communication protocol. We study the expected number of bits communicated among the parties and also exhibit a trade-off between the number of rounds of the rejection algorithm and the number of bits transmitted in the initial phase. Finally, we apply remote sampling to the simulation of quantum entanglement in its essentially most general form possible, when an arbitrary finite number *m* of parties share systems of arbitrary finite dimensions on which they apply arbitrary measurements (not restricted to being projective measurements, but restricted to finitely many possible outcomes). In case the dimension of the systems and the number of possible outcomes per party are bounded by a constant, it suffices to communicate an expected $O(m^{2})$ bits in order to simulate *exactly* the outcomes that these measurements would have produced on those systems.

## 1. Introduction

**Figure 1:**
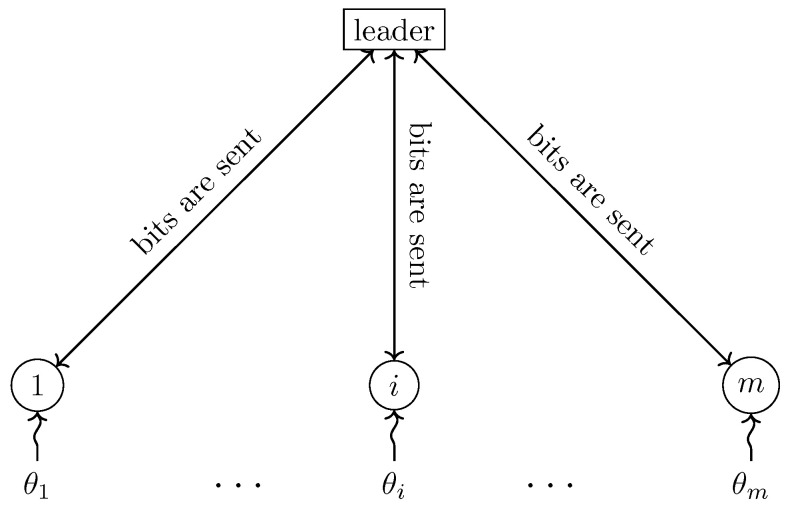
The communication scheme.

Let X be a nonempty finite set containing *n* elements and $p={\left(p_{x}\right)}_{x\in X}$ be a probability vector parameterized by some vector $\theta =\left(\theta_{1},\ldots ,\theta_{m}\right)\in \Theta^{m}$ for an integer m≥2. For instance, the set Θ can be the real interval [0,1] or the set of Hermitian semi-definite positive matrices as it is the case for the simulation of entanglement. The probability vector *p* defines a random variable *X* such that $P\left\{X=x\right\}\;\overset{\mathrm{def}}{=}\;p_{x}$ for x∈X. To sample exactly the probability vector *p* means to produce an output *x* such that $P\left\{X=x\right\}=p_{x}$. The problem of sampling probability distributions has been studied and is still studied extensively within different random and computational models. Here, we are interested in sampling *exactly* a discrete distribution whose defining parameters are distributed among *m* different parties. The $\theta_{i}$’s for i∈{1,…,m} are stored in *m* different locations where the *i*th party holds $\theta_{i}$. In general, any communication topology between the parties would be allowed, but, in this work, we concentrate for simplicity on a model in which we add a designated party known as the *leader*, whereas the *m* other parties are known as the *custodians* because each of them is sole keeper of the corresponding parameter θ—hence there are m+1 parties in total. The leader communicates in both directions with the custodians, who do not communicate among themselves. Allowing inter-custodian communication would not improve the communication efficiency of our scheme and can, at best, halve the number of bits communicated in any protocol. However, it could dramatically improve the sampling *time* in a realistic model in which each party is limited to sending and receiving a fixed number of bits at any given time step, as demonstrated in our previous work [1] concerning a special case of the problem considered here. The communication scheme is illustrated in Figure 1.

It may seem paradoxical that the leader can sample *exactly* the probability vector *p* with a *finite* expected number of bits sent by the custodians, who may hold *continuous* parameters that define *p*. However, this counterintuitive possibility has been known to be achievable for more than a quarter-century in earlier work on the simulation of quantum entanglement by classical communication, starting with Refs. [2,3,4,5,6,7], continuing with Refs. [8,9,10,11,12,13,14], etc. for the bipartite case and Refs. [15,16,17], etc. for the multipartite case, and culminating with our own Ref. [1].

Our protocol to sample remotely a given probability vector is presented in Section 2. For this purpose, the von Neumann rejection algorithm [18] is modified to produce an output x∈X with exact probability $p_{x}$ using mere approximations of those probabilities, which are computed based on partial knowledge of the parameters transmitted on demand by the custodians to the leader. For the sake of simplicity, and to concentrate on the new techniques, we assume initially that algebraic operations on real numbers can be carried out with infinite precision and that continuous random variables can be sampled. Later, in Section 4, we build on techniques developed in Ref. [1] to obtain exact sampling in a realistic scenario in which all computations are performed with finite precision and the only source of randomness comes from flipping independent fair coins.

In the intervening Section 3, we study our motivating application of remote sampling, which is the simulation of quantum entanglement using classical resources and classical communication. Readers who may not be interested in quantum information can still benefit from Section 2 and most of Section 4, which make no reference to quantum theory in order to explain our general remote sampling strategies. A special case of remote sampling has been used by the authors [1], in which the aim was to sample a specific probability distribution appearing often in quantum information science, namely the *m*-partite Greenberger–Horne–Zeilinger (GHZ) distribution [19]. More generally, consider a quantum system of dimension $d=d_{1}\cdots d_{m}$ represented by a density matrix ρ known by the leader (surprisingly, the custodians have no need to know ρ). Suppose that there are *m* generalized measurements (povms) acting on quantum systems of dimensions $d_{1},\ldots ,d_{m}$ whose possible outcomes lie in sets $X_{1},\ldots ,X_{m}$ of cardinality $n_{1},\ldots ,n_{m}$, respectively. Each custodian knows one and only one of the povms and nothing else about the others. The leader does not know initially any information about any of the povms. Suppose in addition that the leader can generate independent identically distributed uniform random variables on the real interval [0,1]. We show how to generate a random vector $X=\left(X_{1},\ldots ,X_{m}\right)\in X=X_{1}\times \ldots \times X_{m}$ sampled from the exact joint probability distribution that would be obtained if each custodian *i* had the *i*th share of ρ (of dimension $d_{i}$) and measured it according to the *i*th povm, producing outcome $x_{i}\in X_{i}$. This task is defined formally in Section 3, where we prove that the total expected number of bits transmitted between the leader and the custodians using remote sampling is $O(m^{2})$ provided all the $d_{i}$’s and $n_{i}$’s are bounded by some constant. The exact formula, involving *m* as well as the $d_{i}$’s and $n_{i}$’s, is given as Equation (14) in Section 3, where *d* and *n* denote the product of the $d_{i}$’s and the $n_{i}$’s, respectively. In Section 4, we obtain the same asymptotic result in the more realistic scenario in which the only source of randomness comes from independent identically distributed uniform random *bits*. This result subsumes that of Ref. [1] since all $d_{i}$’s and $n_{i}$’s are equal to 2 for projective measurements on individual qubits of the *m*-partite GHZ state.

## 2. Remote Sampling

As explained in the Introduction, we show how to sample *remotely* a discrete probability vector $p={\left(p_{x}\right)}_{x\in X}$. The task of sampling is carried by a *leader* ignorant of some parameters $\theta =(\theta_{1},\ldots ,\theta_{m})$ that come in the definition of the probability vector, where each $\theta_{i}$ is known by the *i*th *custodian* only, with whom the leader can communicate. We strive to minimize the amount of communication required to achieve this task.

To solve our conundrum, we modify the von Neumann rejection algorithm [18,20]. Before explaining those modifications, let us review the original algorithm. Let $q={\left(q_{x}\right)}_{x\in X}$ be a probability vector that we know how to sample on the same set X, and let C≥1 be such that $p_{x}\leq Cq_{x}$ for all x∈X. The classical von Neumann rejection algorithm is shown as Algorithm 1. It is well known that the expected number of times round the **repeat** loop is exactly *C*.

**Algorithm 1** Original von Neumann rejection algorithm
1:
**repeat**
2:   Sample *X* according to ${\left(q_{x}\right)}_{x\in X}$3:   Sample *U* uniformly on [0,1]4:   **if** $UCq_{X}\leq p_{X}$ **then**5:     **return** *X* {*X* is accepted}6:  **end if**7:
**end repeat**



If only partial knowledge about the parameters defining *p* is known, it would seem that the condition in line 4 cannot be decided. Nevertheless, the strategy is to build a sequence of increasingly accurate approximations that converge to the left and right sides of the test. As explained below, the number of bits transmitted depends on the number of bits needed to compute *q*, and on the accuracy in *p* required to accept or reject. This task can be achieved either in the *random bit model*, in which only i.i.d. random bits are generated, or in the less realistic *uniform model*, in which uniform continuous random variables are needed. The random bit model was originally suggested by von Neumann [18], but only later given this name and formalized by Knuth and Yao [21]. In this section, we concentrate for simplicity on the uniform model, leaving the more practical random bit model for Section 4.

**Definition** **1.** *A t-bit approximation of a real number x is any $\hat{x}$ such that $|x-\hat{x}|\leq 2^{-t}$. A special case of t-bit approximation is the t-bit truncation $\hat{x}=\mathrm{sign}\left(x\right)\lfloor |x|2^{t}\rfloor /2^{t}$, where sign(x) is equal to +1, 0 or −1 depending on the sign of x. If α=a+bi is a complex number, where $i=\sqrt{-1}$, then a t-bit approximation (resp. truncation) $\hat{\alpha }$ of α is any $\hat{a}+\hat{b}i$, where $\hat{a}$ and $\hat{b}$ are t-bit approximations (resp. truncations) of a and b, respectively.*

Note that we assume without loss of generality that approximations of probabilities are always constrained to be real numbers between 0 and 1, which can be enforced by snapping any out-of-bound approximation (even if it is a complex number) to the closest valid value.

Consider an integer $t_{0}>0$ to be determined later. Our strategy is for the leader to compute the probability vector $q={\left(q_{x}\right)}_{x\in X}$ defined below, based on $t_{0}$-bit approximations $p_{x}\left(t_{0}\right)$ of the probabilities $p_{x}$ for each x∈X. For this purpose, the leader receives sufficient information from the custodians to build the entire vector *q* at the outset of the protocol. This makes *q* the “easy-to-sample” distribution required in von Neumann’s technique, which is easy not from a computational viewpoint, but in the sense that no further communication is required for the leader to sample it as many times as needed.

Let

(1) $$C=\sum_{x}\left(p_{x}\left(t_{0}\right)+2^{-t_{0}}\right)$$

and

(2) $$q_{x}=\left(p_{x}\left(t_{0}\right)+2^{-t_{0}}\right)/C\;.$$

Noting that $\sum_{x}q_{x}=1$, these $q_{x}$ define a proper probability vector $q={\left(q_{x}\right)}_{x\in X}$. Using the definition of a *t*-bit approximation and the definition of $q_{x}$ from Equation (2), we have that

$$p_{x}\leq \left(p_{x}\left(t_{0}\right)+2^{-t_{0}}=Cq_{x}\right)\leq p_{x}+2\times 2^{-t_{0}}\;.$$

Taking the sum over the possible values for *x* and recalling that set X is of cardinality *n*,

(3) $$1\leq C\leq 1+2^{1-t_{0}}n\;.$$

Consider any x∈X sampled according to *q* and *U* sampled uniformly in [0,1] as in lines 2 and 3 of Algorithm 1. Should *x* be accepted because $UCq_{x}\leq p_{x}$, this can be certified by any *t*-bit approximation $p_{x}\left(t\right)$ of $p_{x}$ such that $UCq_{x}\leq p_{x}\left(t\right)-2^{-t}$ for some positive integer *t* since $p_{x}\left(t\right)\leq p_{x}+2^{-t}$. Conversely, any integer *t* such that $UCq_{x}>p_{x}\left(t\right)+2^{-t}$ certifies that *x* should be rejected because it implies that $UCq_{x}>p_{x}$ since $p_{x}\left(t\right)\geq p_{x}-2^{-t}$. On the other hand, no decision can be made concerning $UCq_{x}$ versus $p_{x}$ if $-2^{-t}<UCq_{x}-p_{x}\left(t\right)\leq 2^{-t}$. It follows that one can modify Algorithm 1 above into Algorithm 2 below, in which a sufficiently precise approximation of $p_{x}$ suffices to make the correct decision to accept or reject an *x* sampled according to distribution *q*. A well-chosen value of $t_{0}$ must be input into this algorithm, as discussed later.

**Algorithm 2** Modified rejection algorithm—Protocol for the leader**Input:** Value of $t_{0}$
1:Compute $p_{x}\left(t_{0}\right)$ for each x∈X{The leader needs information from the custodians in order to compute these approximations}2:Compute *C* and $q={\left(q_{x}\right)}_{x\in X}$ as per Equations (1) and (2)3:Sample *X* according to *q*4:Sample *U* uniformly on [0,1]5:**for**$t=t_{0}$ ∞ **do**6:   **if** $UCq_{X}\leq p_{X}\left(t\right)-2^{-t}$ **then**7:     **return** *X* {*X* is accepted}8:   **else if** $UCq_{X}>p_{X}\left(t\right)+2^{-t}$ **then**9:     **go to line** 3 {*X* is rejected}10:  **else**11:    Continue the **for** loop    {We cannot decide whether to accept or reject because $-2^{-t}<UCq_{x}-p_{x}\left(t\right)\leq 2^{-t}$;     communication may be required in order for the leader to compute $p_{X}\left(t+1\right)$;     it could be that bits previously communicated to compute $p_{x}\left(t\right)$ can be reused.}12:   **end if**13:
**end for**

**Theorem** **1.** *Algorithm 2 is correct, i.e., it terminates and returns X=x with probability $p_{x}$. Furthermore, let T be the random variable that denotes the value of variable t upon termination of any instance of the* **for** *loop, whether the loop terminates in rejection or acceptation. Then,*

(4) $$E\left(T\right)\leq t_{0}+3\;.$$

**Proof.**  Consider any x∈X and $t\geq t_{0}$. To reach T>t, it must be that $-2^{-t}<UCq_{x}-p_{x}\left(t\right)\leq 2^{-t}$. Noting that $q_{x}\neq 0$ according to Equation (2), the probability that T>t when X=x is therefore upper-bounded as follows:

(5) $$\begin{array}{cc} P\;\left\{T>t\;|\;X=x\right\} & \leq P\;\left\{-2^{-t}<UCq_{x}-p_{x}\left(t\right)\leq 2^{-t}\right\}\\ & =P\;\left\{\frac{p_{x}\left(t\right)-2^{-t}}{Cq_{x}}<U\leq \frac{p_{x}\left(t\right)+2^{-t}}{Cq_{x}}\right\}\\ & \leq \frac{p_{x}\left(t\right)+2^{-t}}{Cq_{x}}-\frac{p_{x}\left(t\right)-2^{-t}}{Cq_{x}}=\frac{2\times 2^{-t}}{Cq_{x}}\leq 2^{t_{0}-t+1}\;. \end{array}$$

The last inequality uses the fact that

$$Cq_{x}=p_{x}\left(t_{0}\right)+2^{-t_{0}}\geq 2^{-t_{0}}\;.$$

It follows that the probability that more turns round the **for** loop are required decreases exponentially with each new turn once $t>t_{0}+1$, which suffices to guarantee termination of the **for** loop with probability 1. Termination of the algorithm itself comes from the fact that the choice of *X* and *U* in lines 3 and 4 leads to acceptance at line 7—and therefore termination—with probability 1/C, as demonstrated by von Neumann in the analysis of his rejection algorithm.

The fact that X=x is returned with probability $p_{x}$ is an immediate consequence of the correctness of the von Neumann rejection algorithm since our adaptation of this method to handle the fact that only approximations of $p_{X}$ are available does not change the decision to accept or reject any given candidate sampled according to *q*.

In order to bound the expectation of *T*, we note that P{T>t|X=x}=1 when $t<t_{0}$ since we start the **for** loop at $t=t_{0}$. We can also use vacuous $P\{T>t_{0}\;|\;X=x\}\leq 1$ rather than the worse-than-vacuous upper bound of 2 given by Equation (5) in the case $t=t_{0}$. Therefore,

$$\begin{array}{cc} E(T\;|\;X=x) & =\sum_{t=0}^{\infty }P\{T>t\;|\;X=x\}\\ & =\sum_{t=0}^{t_{0}}P\{T>t\;|\;X=x\}+\;\;\;\;\sum_{t=t_{0}+1}^{\infty }\;\;P\{T>t\;|\;X=x\}\\ & \leq t_{0}+1+2^{t_{0}+1}\;\;\;\;\sum_{t=t_{0}+1}^{\infty }\;\;2^{-t}=t_{0}+3\;. \end{array}$$

It remains to note that, since $E\left(T\;\right|\;X=x)\leq t_{0}+3$ for all x∈X, it follows that $E\left(T\right)\leq t_{0}+3$ without condition. □

Let *S* be the random variable that represents the number of times variable *X* is sampled according to *q* at line 3, and let $T_{i}$ be the random variable that represents the value of variable *T* upon termination of the *i*th instance of the **for** loop starting at line 5, for i∈{1,…,S}. The random variables $T_{i}$ are independently and identically distributed as the random variable *T* in Theorem 1 and the expected value of *S* is *C*. Let $X_{1},\ldots ,X_{S}$ be the random variables chosen at successive passes at line 3, so that $X_{1},\ldots ,X_{S-1}$ are rejected, whereas $X_{S}$ is returned as the final result of the algorithm.

To analyse the communication complexity of Algorithm 2, we introduce function $\gamma_{x}\left(t\right)$ for each x∈X and $t>t_{0}$, which denotes the *incremental* number of bits that the leader must receive from the custodians in order to compute $p_{x}\left(t\right)$, taking account of the information that may already be available if he had previously computed $p_{x}\left(t-1\right)$. For completeness, we include in $\gamma_{x}\left(t\right)$ the cost of the communication required for the leader to request more information from the custodians. We also introduce function δ(t) for t≥0, which denotes the number of bits that the leader must receive from the custodians in order to compute $p_{x}\left(t\right)$ for all x∈X in a “simultaneous” manner. Note that it could be much less expensive to compute those *n* values than *n* times the cost of computing any single one of them because some of the parameters held by the custodians may be relevant to more than one of the $p_{x}$’s. The total number of bits communicated in order to implement Algorithm 2 is therefore given by random variable

$$Z=\delta \left(t_{0}\right)+\sum_{i=1}^{S}\;\sum_{t=t_{0}+1}^{T_{i}}\gamma_{X_{i}}\left(t\right)\;.$$

For simplicity, let us define function $\gamma \left(t\right)\;\overset{\mathrm{def}}{=}\;\max_{x\in X}\gamma_{x}\left(t\right)$. We then have

$$Z\leq \delta \left(t_{0}\right)+\sum_{i=1}^{S}\;\sum_{t=t_{0}+1}^{T_{i}}\gamma (t)\;,$$

whose expectation, according to Wald’s identity, is

(6) $$E\left(Z\right)\leq \delta \left(t_{0}\right)+E\left(S\right)\;E\left(\sum_{t=t_{0}+1}^{T}\gamma (t)\right)\;.$$

Assuming the value of γ(t) is upper-bounded by some γ,

(7) $$\begin{array}{cc} E(Z) & \leq \delta \left(t_{0}\right)+E\left(S\right)E\left(T-t_{0}\right)\gamma \\ & \leq \delta (t_{0})+3\gamma C\\ & \leq \delta \left(t_{0}\right)+3\gamma \left(1+2^{1-t_{0}}n\right) \end{array}$$

because E(S)=C and using Equations (4) and (3).

Depending on the specific application, which determines γ and function δ(t), Equation (7) is key to a trade-off that can lead to an optimal choice of $t_{0}$ since a larger $t_{0}$ decreases $2^{1-t_{0}}$ but is likely to increase $\delta (t_{0})$. The value of γ may play a rôle in the balance. The next section, in which we consider the simulation of quantum entanglement by classical communication, gives an example of this trade-off in action.

## 3. Simulation of Quantum Entanglement Based on Remote Sampling

Before introducing the simulation of entanglement, let us establish some notation and mention the mathematical objects that we shall need. It is assumed that the reader is familiar with linear algebra, in particular the notion of a semi-definite positive matrix, Hermitian matrix, trace of a matrix, tensor product, etc. For a discussion about the probabilistic and statistical nature of quantum theory, see Ref. [22]. For convenience, we use [n] to denote the set {1,2,…,n} for any integer *n*.

Consider integers $m,d_{1},d_{2},\ldots ,d_{m},n_{1},n_{2},\ldots ,n_{m}$, all greater than or equal to 2. Define $d=\prod_{i=1}^{m}d_{i}$ and $n=\prod_{i=1}^{m}n_{i}$. Let ρ be a d×d density matrix. Recall that any density matrix is Hermitian, semi-definite positive and unit-trace, which implies that its diagonal elements are real numbers between 0 and 1. For each i∈[m] and $j\in [n_{i}]$, let $M_{ij}$ be a $d_{i}\times d_{i}$ Hermitian semi-definite positive matrix such that

(8) $$\sum_{j\in [n_{i}]}M_{ij}=I_{d_{i}}\;,$$

where $I_{d_{i}}$ is the $d_{i}\times d_{i}$ identity matrix. In other words, each set ${\left\{M_{ij}\right\}}_{j\in [n_{i}]}$ is a povm (positive-operator valued measure) [22].

As introduced in Section 1, we consider one *leader* and *m* *custodians*. The leader knows density matrix ρ and the *i*th custodian knows the *i*th povm, meaning that he knows the matrices $M_{ij}$ for all $j\in [n_{i}]$. If a physical system of dimension *d* in state ρ were shared between the custodians, in the sense that the *i*th custodian had possession of the *i*th subsystem of dimension $d_{i}$, each custodian could perform locally his assigned povm and output the outcome, an integer between 1 and $n_{i}$. The joint output would belong to $X\;\;\overset{\mathrm{def}}{=}\;\;\left[n_{1}\right]\times \left[n_{2}\right]\times \cdots \times \left[n_{m}\right]$, a set of cardinality *n*, sampled according to the probability distribution stipulated by the laws of quantum theory, which we review below.

Our task is to sample X with the exact same probability distribution even though there is no physical system in state ρ available to the custodians, and in fact all parties considered are purely classical! We know from Bell’s Theorem [23] that this task is impossible in general without communication, even when m=2, and our goal is to minimize the amount of communication required to achieve it. Special cases of this problem have been studied extensively for expected [1,2,4,5,6], etc. and worst-case [3,8], etc. communication complexity, but here we solve it in its essentially most general setting, albeit only in the expected sense. For this purpose, the leader will centralize the operations while requesting as little information as possible from the custodians on their assigned povms. Once the leader has successfully sampled $X=(X_{1},\ldots ,X_{m})$, he transmits each $X_{i}$ to the *i*th custodian, who can then output it as would have been the case had quantum measurements actually taken place.

We now review the probability distribution X that we need to sample, according to quantum theory. For each vector $x=(x_{1},\ldots ,x_{m})\in X$, let $M_{x}$ be the d×d tensor product of matrices $M_{ix_{i}}$ for each i∈[m]:

(9) $$M_{x}=\overset{m}{\underset{i=1}{⨂}}M_{ix_{i}}\;.$$

The set ${\left\{M_{x}\right\}}_{x\in X}$ forms a global povm of dimension *d*, which applied to density matrix ρ defines a joint probability vector on X. The probability $p_{x}$ of obtaining any $x=(x_{1},\ldots ,x_{m})\in X$ is given by

(10) $$p_{x}=\mathrm{Tr}\left(\rho M_{x}\right)=\mathrm{Tr}\left(\rho \left(\overset{m}{\underset{i=1}{⨂}}M_{ix_{i}}\right)\right)\;.$$

For a matrix *A* of size s×s and any pair of indices *r* and *c* between 0 and s−1, we use ${\left(A\right)}_{rc}$ to denote the entry of *A* located in the $r^{\mathrm{th}}$ row and $c^{\mathrm{th}}$ column. Matrix indices start at 0 rather than 1 to facilitate Fact 2 below. We now state various facts for which we provide cursory justifications since they follow from elementary linear algebra and quantum theory, or they are lifted from previous work.

**Fact** **1.** For all x∈X, we have $0\leq p_{x}\leq 1$ when $p_{x}$ is defined according to Equation (10); furthermore, $\sum_{x\in X}p_{x}=1$. This is obvious because quantum theory tells us that Equation (10) defines a probability distribution over all possible outcomes x∈X, as sampled by the joint measurement. Naturally, this statement could also be proven from Equations (8) and (10) using elementary linear algebra.

**Fact** **2.** For each $x=(x_{1},\ldots ,x_{m})\in X$, matrix $M_{x}$ is the tensor product of *m* matrices as given in Equation (9). Therefore, each entry ${\left(M_{x}\right)}_{rc}$ is the product of *m* entries of the $M_{ix_{i}}$’s. Specifically, consider any indices *r* and *c* between 0 and d−1 and let $r_{i}$ and $c_{i}$ be the indices between 0 and $d_{i}-1$, for each i∈[m], such that

$$\begin{array}{cc} r & =r_{1}+r_{2}d_{1}+r_{3}d_{1}d_{2}+\ldots +r_{m}d_{1}\cdots d_{m-1},\\ c & =c_{1}+c_{2}d_{1}+c_{3}d_{1}d_{2}+\ldots +c_{m}d_{1}\cdots d_{m-1}\;. \end{array}$$

The $r_{i}$’s and $c_{i}$’s are uniquely defined by the principle of mixed-radix numeration. We have

$${\left(M_{x}\right)}_{rc}=\prod_{i=1}^{m}{\left(M_{ix_{i}}\right)}_{r_{i}c_{i}}\;.$$

**Fact** **3.** Let *M* be a Hermitian semi-definite positive matrix. Every entry ${\left(M\right)}_{ij}$ of the matrix satisfies

$${|\left(M\right)}_{ij}|\leq \sqrt{{\left(M\right)}_{ii}{\left(M\right)}_{jj}}\;.$$

This follows from the fact that all principal submatrices of any Hermitian semi-definite positive matrix are semi-definite positive [24] (Observation 7.1.2, page 430). In particular, the principal submatrix

$$\left(\begin{array}{cc} {\left(M\right)}_{ii} & {\left(M\right)}_{ij}\\ {\left(M\right)}_{ji} & {\left(M\right)}_{jj} \end{array}\right)$$

is semi-definite positive, and therefore it has nonnegative determinant:

$${\left(M\right)}_{ii}{\left(M\right)}_{jj}-{\left(M\right)}_{ij}{\left(M\right)}_{ji}={\left(M\right)}_{ii}{\left(M\right)}_{jj}-{\left(M\right)}_{ij}{\left(M\right)}_{ij}^{*}={\left(M\right)}_{ii}{\left(M\right)}_{jj}-{\left|{\left(M\right)}_{ij}\right|}^{2}\geq 0$$

by virtue of *M* being Hermitian, where $\alpha^{*}$ denotes the complex conjugate of *α*.

**Fact** **4.** The norm ${|\left(\rho \right)}_{ij}|$ of any entry of a density matrix *ρ* is less than or equal to 1. This follows directly from Fact 3 since density matrices are Hermitian semi-definite positive, and from the fact that diagonal entries of density matrices, such as ${\left(\rho \right)}_{ii}$ and ${\left(\rho \right)}_{jj}$, are real values between 0 and 1.

**Fact** **5.** Given any povm ${\left\{M_{\ell }\right\}}_{\ell =1}^{L}$, we have that

1. $0\leq {\left(M_{\ell }\right)}_{ii}\leq 1$ for all *ℓ* and *i*, and
2. $|{\left(M_{\ell }\right)}_{ij}|\leq 1$ for all *ℓ*, *i* and *j*.

The first statement follows from the fact that $\sum_{\ell =1}^{L}M_{\ell }$ is the identity matrix by definition of povms, and therefore $\sum_{\ell =1}^{L}{\left(M_{\ell }\right)}_{ii}=1$ for all *i*, and the fact that each ${\left(M_{\ell }\right)}_{ii}\geq 0$ because each $M_{\ell }$ is semi-definite positive. The second statement follows from the first by applying Fact 3.

**Fact** **6.** (This is a special case of Theorem 1 from Ref. [1], with v=0). Let k≥1 be an integer and consider any two real numbers *a* and *b*. If $\hat{a}$ and $\hat{b}$ are arbitrary *k*-bit approximations of *a* and *b*, respectively, then $\hat{a}+\hat{b}$ is a (k−1)-bit approximation of a+b. If, in addition, *a* and *b* are known to lie in interval [−1,1], which can also be assumed without loss of generality concerning $\hat{a}$ and $\hat{b}$ since otherwise they can be safely pushed back to the appropriate frontier of this interval, then $\hat{a}\hat{b}$ is a (k−1)-bit approximation of ab.

**Fact** **7.** Let k≥1 be an integer and consider any two *complex* numbers *α* and *β*. If $\hat{\alpha }$ and $\hat{\beta }$ are arbitrary *k*-bit approximations of *α* and *β*, respectively, then $\hat{\alpha }+\hat{\beta }$ is a (k−1)-bit approximation of α+β. If, in addition, k≥2 and the real and imaginary parts of *α* and *β* are known to lie in interval [−1,1], which can also be assumed without loss of generality concerning $\hat{\alpha }$ and $\hat{\beta }$, then $\hat{\alpha }\hat{\beta }$ is a (k−2)-bit approximation of αβ. This is a direct consequence of Fact 6 in the case of addition. In the case of multiplication, consider α=a+bi, β=c+di, $\hat{\alpha }=\hat{a}+\hat{b}i$ and $\hat{\beta }=\hat{c}+\hat{d}i$, so that

$$\alpha \beta =\left(ac-bd\right)+\left(ad+bc\right)i\;\;\;\mathrm{and}\;\;\;\hat{\alpha }\hat{\beta }=\left(\hat{a}\hat{c}-\hat{b}\hat{d}\right)+\left(\hat{a}\hat{d}+\hat{b}\hat{c}\right)i\;.$$

By the multiplicative part of Fact 6, $\hat{a}\hat{c}$, $\hat{b}\hat{d}$, $\hat{a}\hat{d}$ and $\hat{b}\hat{c}$ are (k−1)-bit approximations of ac, bd, ad and bc, respectively; and then by the additive part of the same fact (which obviously applies equally well to subtraction), $\hat{a}\hat{c}-\hat{b}\hat{d}$ and $\hat{a}\hat{d}+\hat{b}\hat{c}$ are (k−2)-bit approximations of ac−bd and ad+bc, respectively.

**Fact** **8** (This is Corollary 2 from Ref. [1]). Let m≥2 and k≥⌈lgm⌉ be integers and let ${\left\{a_{j}\right\}}_{j=1}^{m}$ and ${\left\{{\hat{a}}_{j}\right\}}_{j=1}^{m}$ be real numbers and their *k*-bit approximations, respectively, all in interval [−1,1]. Then, $\prod_{j=1}^{m}{\hat{a}}_{j}$ is a (k−⌈lgm⌉)-bit approximation of $\prod_{j=1}^{m}a_{j}$.

**Fact** **9.** Let m≥2 and k≥2⌈lgm⌉ be integers and let ${\left\{\alpha_{j}\right\}}_{j=1}^{m}$ and ${\left\{{\hat{\alpha }}_{j}\right\}}_{j=1}^{m}$ be complex numbers and their *k*-bit approximations, respectively. Provided it is known that $|\alpha_{j}|\leq 1$ for each j∈[m], a (k−2⌈lgm⌉)-bit approximation of $\prod_{j=1}^{m}\alpha_{j}$ can be computed from knowledge of the ${\hat{\alpha }}_{j}$’s. The proof of this fact follows essentially the same template as Fact 8, except that *two* bits of precision may be lost at each level up the binary tree introduced in Ref. [1], due to the difference between Facts 6 and 7. A subtlety occurs in the need for Fact 7 to apply that the real and imaginary parts of all the complex numbers under consideration must lie in interval [−1,1]. This is automatic for the exact values since the $\alpha_{j}$’s are upper-bounded in norm by 1 and the product of such-bounded complex numbers is also upper-bounded in norm by 1, which implies that their real and imaginary parts lie in interval [−1,1]. For the approximations, however, we cannot force their *norm* to be bounded by 1 because we need the approximations to be rational for communication purposes. Fortunately, we can force the real and imaginary parts of all approximations computed at each level up the binary tree to lie in interval [−1,1] because we know that they approximate such-bounded numbers. Note that the product of two complex numbers whose real and imaginary parts lie in interval [−1,1], such as $1+2^{-k}i$ and $1-2^{-k}i$, may not have this property, even if they are *k*-bit approximations of numbers bounded in norm by 1.

**Fact** **10.** Let s≥2 and k≥⌈lgs⌉ be integers and let ${\left\{\alpha_{j}\right\}}_{j=1}^{s}$ and ${\left\{{\hat{\alpha }}_{j}\right\}}_{j=1}^{s}$ be complex numbers and their *k*-bit approximations, respectively, without any restriction on their norm. Then $\sum_{j=1}^{s}{\hat{\alpha }}_{j}$ is a (k−⌈lgs⌉)-bit approximation of $\sum_{j=1}^{s}\alpha_{j}$. Again, this follows the same proof template as Fact 8, substituting multiplication of real numbers by addition of complex numbers, which allows us to drop any condition on the size of the numbers considered.

**Fact** **11.** Consider any $x=(x_{1},\ldots ,x_{m})\in X$ and any positive integer *t*. In order to compute a *t*-bit approximation of $p_{x}$, it suffices to have (t+1+⌈2lgd⌉+2⌈lgm⌉)-bit approximations of each entry of the $M_{ix_{i}}$ matrices for all i∈[m]. This is because

(11) $$\begin{array}{cc} p_{x} & =\mathrm{Tr}\left(\rho M_{x}\right)=\sum_{r=0}^{d-1}{\left(\rho M_{x}\right)}_{rr}\\ & =\sum_{r=0}^{d-1}\sum_{c=0}^{d-1}{\left(\rho \right)}_{rc}{\left(M_{x}\right)}_{cr}\\ & =\sum_{r=0}^{d-1}\sum_{c=0}^{d-1}{\left(\rho \right)}_{rc}\prod_{i=1}^{m}{\left(M_{ix_{i}}\right)}_{c_{i}r_{i}} \end{array}$$

by virtue of Fact 2. Every term of the double sum in Equation (11) involves a product of *m* entries, one per povm element, and therefore incurs a loss of at most 2⌈lgm⌉ bits of precision by Fact 9, whose condition holds thanks to Fact 5. An additional bit of precision may be lost in the multiplication by ${\left(\rho \right)}_{rc}$, even though that value is available with arbitrary precision (and is upper-bounded by 1 in norm by Fact 4) because of the additions involved in multiplying complex numbers. Then, we have to add $s=d^{2}$ terms, which incurs an additional loss of at most ⌈lgs⌉=⌈2lgd⌉ bits of precision by Fact 10. In total, (t+1+⌈2lgd⌉+2⌈lgm⌉)-bit approximations of the ${\left(M_{ix_{i}}\right)}_{c_{i}r_{i}}$’s will result in a *t*-bit approximation of $p_{x}$.

**Fact** **12.** The leader can compute $p_{x}\left(t\right)$ for any specific $x=(x_{1},\ldots ,x_{m})\in X$ and integer *t* if he receives a total of

$$\left(t+2+\left\lceil 2\mathrm{lg}d\right\rceil +2\left\lceil \mathrm{lg}m\right\rceil \right)\sum_{i=1}^{m}d_{i}^{2}$$

bits from the custodians. This is because the *i*th custodian has the description of matrix $M_{ix_{i}}$ of size $d_{i}\times d_{i}$, which is defined by exactly $d_{i}^{2}$ *real* numbers since the matrix is Hermitian. By virtue of Fact 11, it is sufficient for the leader to have (t+1+⌈2lgd⌉+2⌈lgm⌉)-bit approximations for all those $\sum_{i=1}^{m}d_{i}^{2}$ numbers. Since each one of them lies in interval [−1, 1] by Fact 5, well-chosen *k*-bit approximations (for instance *k*-bit truncations) can be conveyed by the transmission of k+1 bits, one of which carries the sign.

Note that the *t*-bit approximation of $p_{x}$ computed according to Fact 12, say a+bi, may very well have a nonzero imaginary part *b*, albeit necessarily between $-2^{-t}$ and $2^{-t}$. Since $p_{x}\left(t\right)$ must be a real number between 0 and 1, the leader sets $p_{x}\left(t\right)=\max\left(0,\min\left(1,a\right)\right)$, taking no account of *b*, although a paranoid leader may wish to test that $-2^{-t}\leq b\leq 2^{-t}$ indeed and raise an alarm in case it is not (which of course is mathematically impossible unless the custodians are not given proper povms, unless they misbehave, or unless a computation or communication error has occurred).

**Fact** **13.** For any *t*, the leader can compute $p_{x}\left(t\right)$ for each and every x∈X if he receives

$$\delta \left(t\right)\overset{\mathrm{def}}{=}\left(t+2+\left\lceil 2\mathrm{lg}d\right\rceil +2\left\lceil \mathrm{lg}m\right\rceil \right)\sum_{i=1}^{m}n_{i}d_{i}^{2}$$

bits from the custodians. This is because it suffices for each custodian *i* to send to the leader (t+1+⌈2lgd⌉+2⌈lgm⌉)-bit approximations of all $n_{i}d_{i}^{2}$ real numbers that define the entire *i*th povm, i.e., all the matrices $M_{ij}$ for $j\in [n_{i}]$. This is a nice example of the fact that it may be much less expensive for the leader to compute at once $p_{x}\left(t\right)$ for all x∈X, rather than computing them one by one independently, which would cost

$$n\left(t+2+\left\lceil 2\mathrm{lg}d\right\rceil +2\left\lceil \mathrm{lg}m\right\rceil \right)\sum_{i=1}^{m}d_{i}^{2}=\left(t+2+\left\lceil 2\mathrm{lg}d\right\rceil +2\left\lceil \mathrm{lg}m\right\rceil \right)\sum_{i=1}^{m}nd_{i}^{2}\gg \delta \left(t\right)$$

bits of communication by applying *n* times Fact 12.

After all these preliminaries, we are now ready to adapt the general template of Algorithm 2 to our entanglement-simulation conundrum, yielding Algorithm 3. We postpone the choice of $t_{0}$ until after the communication complexity analysis of this new algorithm.

**Algorithm 3** Protocol for simulating arbitrary entanglement subjected to arbitrary measurements
1:Each custodian i∈[m] sends his value of $n_{i}$ to the leader, who computes $n=\prod_{i=1}^{m}n_{i}$2:The leader chooses $t_{0}$ and informs the custodians of its value3:Each custodian i∈[m] sends to the leader $\left(t_{0}+1+\left\lceil 2\mathrm{lg}d\right\rceil +2\left\lceil \mathrm{lg}m\right\rceil \right)$-bit truncationsof the real and imaginary parts of the entries defining matrix $M_{ij}$ for each $j\in [n_{i}]$4:The leader computes $p_{x}\left(t_{0}\right)$ for every x∈X, using Fact 135:The leader computes *C* and $q={\left(q_{x}\right)}_{x\in X}$ as per Equations (1) and (2)6:
accept←false
7:
**repeat**
8:   reject←false
9:   The leader samples $X=(X_{1},X_{2},\ldots ,X_{m})$ according to *q*10:   The leader informs each custodian i∈[m] of the value of $X_{i}$11:   The leader samples *U* uniformly on [0,1]12:   $t\leftarrow t_{0}$
13:   **repeat**14:      **if** $UCq_{X}\leq p_{X}\left(t\right)-2^{-t}$ **then**15:         accept←true {*X* is accepted}16:      **else if** $UCq_{X}>p_{X}\left(t\right)+2^{-t}$ **then**17:         reject←true {*X* is rejected}18:      **else**19:         The leader asks each custodian i∈[m] for one more bit in the truncation          of the real and imaginary parts of the entries defining matrix $M_{iX_{i}}$;20:         Using this information, the leader updates $p_{X}\left(t\right)$ into $p_{X}\left(t+1\right)$;21:         t←t+1
22:      **end if**23:   **until** accept **or** reject24:**until** accept25:The leader requests each custodian i∈[m] to output his $X_{i}$


To analyse the expected number of bits of communication required by this algorithm, we apply Equation (7) from Section 2 after defining explicitly the cost parameters $\delta (t_{0})$ for the initial computation of $p_{x}\left(t_{0}\right)$ for all x∈X at lines 3 and 4, and γ for the upgrade from a specific $p_{X}\left(t\right)$ to $p_{X}\left(t+1\right)$ at lines 19 and 20. For simplicity, we shall ignore the negligible amount of communication entailed at line 1 (which is $\sum_{i=1}^{m}\left\lceil \;\mathrm{lg}n_{i}\right\rceil \leq m+\mathrm{lg}n$ bits), line 2 ($\left\lceil \;\mathrm{lg}t_{0}\right\rceil$ bits), line 10 (also $\sum_{i=1}^{m}\left\lceil \;\mathrm{lg}n_{i}\right\rceil$ bits, but repeated $E\left(S\right)\leq 1+2^{1-t_{0}}n$ times) and line 25 (m bits) because they are not taken into account in Equation (7) since they are absent from Algorithm 2. If we counted it all, this would add $O(\left(1+2^{1-t_{0}}n\right)\mathrm{lg}n+\mathrm{lg}t_{0})$ bits to Equation (13) below, which would be less than 10lgn bits added to Equation (14), with no effect at all on Equation (15).

According to Fact 13,

$$\delta \left(t_{0}\right)=\left(t_{0}+2+\left\lceil 2\mathrm{lg}d\right\rceil +2\left\lceil \mathrm{lg}m\right\rceil \right)\sum_{i=1}^{m}n_{i}d_{i}^{2}\;.$$

The cost of line 19 is very modest because we use *truncations* rather than general approximations in line 3 for the leader to compute $p_{x}\left(t_{0}\right)$ for all x∈X. Indeed, it suffices to obtain a single additional bit of precision in the real and imaginary parts of each entry defining matrix $M_{iX_{i}}$ from each custodian i∈[m]. The cost of this update is simply

(12) $$\gamma =m+\sum_{i=1}^{m}d_{i}^{2}$$

bits of communication, where the addition of *m* is to account for the leader needing to request new bits from the custodians. This is a nice example of what we meant by “it could be that bits previously communicated can be reused” in line 11 of Algorithm 2.

Putting it all together in Equation (7), the total expected number of bits communicated in Algorithm 3 in order to sample exactly according to the quantum probability distribution is

(13) $$\begin{array}{cc} E(Z) & \leq \delta \left(t_{0}\right)+3\gamma \left(1+2^{1-t_{0}}n\right)\\ & \leq \left(t_{0}+2+\left\lceil 2\mathrm{lg}d\right\rceil +2\left\lceil \mathrm{lg}m\right\rceil \right)\sum_{i=1}^{m}n_{i}d_{i}^{2}+3\left(1+2^{1-t_{0}}n\right)\left(m+\sum_{i=1}^{m}d_{i}^{2}\right)\;. \end{array}$$

We are finally in a position to choose the value of parameter $t_{0}$. First note that $n=\prod_{i=1}^{m}n_{i}\geq 2^{m}$. Therefore, any constant choice of $t_{0}$ will entail an expected amount of communication that is exponential in *m* because of the right-hand term in Equation (13). At the other extreme, choosing $t_{0}=n$ would also entail an expected amount of communication that is exponential in *m*, this time because of the left-hand term in Equation (13). A good compromise is to choose $t_{0}=\left\lceil \;\mathrm{lg}n\right\rceil$, which results in 1≤C≤3 according to Equation (3), because in that case $2^{t_{0}}\geq n$ and therefore

$$1\leq C\leq 1+2^{1-t_{0}}n=1+\frac{2n}{2^{t_{0}}}\leq 3\;,$$

so that Equation (13) becomes

(14) $$E\left(Z\right)\leq \left(\left\lceil \;\mathrm{lg}n\right\rceil +\left\lceil 2\mathrm{lg}d\right\rceil +2\left\lceil \mathrm{lg}m\right\rceil +2\right)\sum_{i=1}^{m}n_{i}d_{i}^{2}+9\left(m+\sum_{i=1}^{m}d_{i}^{2}\right)\;.$$

In case all the $n_{i}$’s and $d_{i}$’s are upper-bounded by some constant ξ, we have that $n=\prod_{i=1}^{m}n_{i}\leq \xi^{m}$, hence lgn≤mlgξ, similarly lgd≤mlgξ, and also $\sum_{i=1}^{m}n_{i}d_{i}^{2}\leq m\xi^{3}$. It follows that

(15) $$E\left(Z\right)\leq \left(3\xi^{3}\mathrm{lg}\xi \right)m^{2}+O\left(m\log m\right)\;,$$

which is on the order of $m^{2}$, thus matching with our most general method the result that was already known for the very specific case of simulating the quantum *m*-partite GHZ distribution [1].

## 4. Practical Implementation Using a Source of Discrete Randomness

In practice, we cannot work with continuous random variables since our computers have finite storage capacities and finite precision arithmetic. Furthermore, the generation of uniform continuous random variables does not make sense computationally speaking and we must adapt Algorithms 2 and 3 to work in a finite world.

For this purpose, recall that *U* is a uniform continuous random variable on [0,1] used in all the algorithms seen so far. For each i≥1, let $U_{i}$ denote the *i*th bit in the binary expansion of *U*, so that

$$U=0.U_{1}U_{2}\cdots =\sum_{i=1}^{\infty }U_{i}2^{-i}.$$

We acknowledge the fact that the $U_{i}$’s are not uniquely defined in case $U=j/2^{k}$ for integers k>0 and $0<j<2^{k}$, but we only mention this phenomenon to ignore it since it occurs with probability 0 when *U* is uniformly distributed on [0,1]. We denote the *t*-bit truncation of *U* by U[t]:

$$U\left[t\right]\overset{\mathrm{def}}{=}\left\lfloor 2^{t}U\right\rfloor /2^{t}=\sum_{i=1}^{t}U_{i}2^{-i}\;.$$

For all t≥1, we have that

(16) $$U\left[t\right]\leq U<U\left[t\right]+2^{-t}.$$

We modify Algorithm 2 into Algorithm 4 as follows, leaving to the reader the corresponding modification of Algorithm 3, thus yielding a practical protocol for the simulation of general entanglement under arbitrary measurements.

**Algorithm 4** Modified rejection algorithm with discrete randomness source—Protocol for the leader**Input:** Value of $t_{0}$
1:Compute $p_{x}\left(t_{0}\right)$ for each x∈X{The leader needs information from the custodians in order to compute these approximations}2:Compute *C* and $q={\left(q_{x}\right)}_{x\in X}$ as per Equations (1) and (2)3:Sample *X* according to *q*4:
U[0]←0
5:
**for** t=1 **to** $t_{0}-1$ **do**
6:  Generate i.i.d. unbiased bit $U_{t}$7:  $U\left[t\right]\leftarrow U\left[t-1\right]+U_{t}\;2^{-t}$
8:
**end for**
9:**for**$t=t_{0}$ ∞ **do**10:  Generate i.i.d. unbiased bit $U_{t}$11:  $U\left[t\right]\leftarrow U\left[t-1\right]+U_{t}\;2^{-t}$
12:  **if** $\left(U\left[t\right]+2^{-t}\right)Cq_{X}\leq p_{X}\left(t\right)-2^{-t}$ **then**13:    **return** *X* {*X* is accepted}14:  **else if** $U\left[t\right]Cq_{X}>p_{X}\left(t\right)+2^{-t}$ **then**15:    **go to line** 3 {*X* is rejected}16:  **else**17:    Continue the **for** loop    {We cannot decide to accept or reject because $-\left(1+Cq_{X}\right)2^{-t}<U\left[t\right]Cq_{X}-p_{X}\left(t\right)\leq 2^{-t}$; communication may be required in order for the leader to compute $p_{X}\left(t+1\right)$; it could be that bits previously communicated to compute $p_{x}\left(t\right)$ can be reused.}18:  **end if**19:
**end for**

**Theorem** **2.** *Algorithm 4 is correct, i.e., it terminates and returns X=x with probability $p_{x}$. Furthermore, let T be the random variable that denotes the value of variable t upon termination of any instance of the* **for** *loop that starts at line 9, whether it terminates in rejection or acceptation. Then,*

$$E\left(T\right)\leq t_{0}+3+2^{-t_{0}}\;.$$

**Proof.**  This is very similar to the proof of Theorem 1, so let us concentrate on the differences. First note that it follows from Equation (16) and the fact that $|p_{X}\left(t\right)-p_{X}|\leq 2^{-t}$ that

$$\left(U\left[t\right]+2^{-t}\right)Cq_{X}\leq p_{X}\left(t\right)-2^{-t}\Rightarrow UCq_{X}\leq p_{X}\left(t\right)-2^{-t}\Rightarrow UCq_{X}\leq p_{X}$$

and

$$U\left[t\right]Cq_{X}>p_{X}\left(t\right)+2^{-t}\Rightarrow UCq_{X}>p_{X}\left(t\right)+2^{-t}\Rightarrow UCq_{X}>p_{X}\;.$$

Therefore, whenever *X* is accepted at line 13 (resp. rejected at line 15), it would also have been accepted (resp. rejected) in the original von Neumann algorithm, which shows sampling correctness. Conversely, whenever we reach a value of $t\geq t_{0}$ such that $\left(U\left[t\right]+2^{-t}\right)Cq_{X}>p_{X}\left(t\right)-2^{-t}$ and $U\left[t\right]Cq_{X}\leq p_{X}\left(t\right)+2^{-t}$, we do not have enough information to decide whether to accept or reject, and therefore we reach line 17, causing *t* to increase. This happens precisely when

$$-\left(1+Cq_{X}\right)2^{-t}<U\left[t\right]Cq_{X}-p_{X}\left(t\right)\leq 2^{-t}\;.$$

To obtain an upper bound on E(T), we mimic the proof of Theorem 1, but in the discrete rather than continuous regime. In particular, for any x∈X and $t\geq t_{0}$,

(17) $$\begin{array}{cc} P\;\left\{T>t\;|\;X=x\right\} & \leq P\;\left\{-\left(1+Cq_{x}\right)2^{-t}<U\left[t\right]Cq_{x}-p_{x}\left(t\right)\leq 2^{-t}\right\}\\ & =P\;\left\{p_{x}\left(t\right)-\left(1+Cq_{x}\right)2^{-t}<U\left[t\right]Cq_{x}\leq p_{x}\left(t\right)+2^{-t}\right\}\\ & =P\;\left\{\frac{2^{t}p_{x}\left(t\right)}{Cq_{x}}-\frac{1+Cq_{x}}{Cq_{x}}<2^{t}U\left[t\right]\leq \frac{2^{t}p_{x}\left(t\right)}{Cq_{x}}+\frac{1}{Cq_{x}}\right\}\\ & \leq \left[\left(\frac{2^{t}p_{x}\left(t\right)}{Cq_{x}}+\frac{1}{Cq_{x}}\right)-\left(\frac{2^{t}p_{x}\left(t\right)}{Cq_{x}}-\frac{1+Cq_{x}}{Cq_{x}}\right)+1\right]2^{-t} \end{array}$$

(18) $$\begin{array}{c} =2\left(1+\frac{1}{Cq_{x}}\right)2^{-t}\leq 2^{t_{0}-t+1}+2^{1-t}\;\;\;\;\;\;\left(\mathrm{because}\;Cq_{x}\geq 2^{-t_{0}}\right)\;. \end{array}$$

To understand Equation (17), think of $2^{t}U\left[t\right]$ as an integer chosen randomly and uniformly between 0 and $2^{t}-1$. The probability that it falls within some real interval (a,b] for a<b is equal to $2^{-t}$ times the number of integers between 0 and $2^{t}-1$ in that interval, the latter being upper-bounded by the number of unrestricted integers in that interval, which is at most b−a+1.

Noting how similar Equation (18) is to the corresponding Equation (5) in the analysis of Algorithm 2, it is not surprising that the expected value of *T* will be similar as well. Indeed, continuing as in the proof of Theorem 1, without belabouring the details,

(19) $$\begin{array}{cc} E(T\;|\;X=x) & =\sum_{t=0}^{\infty }P\{T>t\;|\;X=x\}\\ & =\sum_{t=0}^{t_{0}+1}P\{T>t\;|\;X=x\}+\;\;\;\;\sum_{t=t_{0}+2}^{\infty }\;\;P\{T>t\;|\;X=x\}\\ & \leq t_{0}+2+2^{t_{0}+1}\;\;\;\;\sum_{t=t_{0}+2}^{\infty }\;\;2^{-t}+2\;\;\;\;\sum_{t=t_{0}+2}^{\infty }\;\;2^{-t}=t_{0}+3+2^{-t_{0}}\;. \end{array}$$

We conclude that $E\left(T\right)\leq t_{0}+3+2^{-t_{0}}$ without condition since Equation (19) does not depend on *x*. □

The similarity between Theorems 1 and 2 means that there is no significant additional cost in the amount of communication required to achieve remote sampling in the random bit model. i.e., if we consider a realistic scenario in which the only source of randomness comes from i.i.d. unbiased bits, compared to an unrealistic scenario in which continuous random variables can be drawn. For instance, the reasoning that led to Equation (7) applies *mutatis mutandis* to conclude that the expected number *Z* of bits that needs to be communicated to achieve remote sampling in the random bit model is

$$E\left(Z\right)\leq \delta \left(t_{0}\right)+\left(3+2^{-t_{0}}\right)\left(1+2^{1-t_{0}}n\right)\gamma \;,$$

where δ and γ have the same meaning as in Section 2.

If we use the random bit approach for the general simulation of quantum entanglement studied in Section 3, choosing $t_{0}=\left\lceil \;\mathrm{lg}n\right\rceil$ again, Equation (14) becomes

(20) $$E\left(Z\right)\leq \left(\left\lceil \;\mathrm{lg}n\right\rceil +\left\lceil 2\mathrm{lg}d\right\rceil +2\left\lceil \mathrm{lg}m\right\rceil +2\right)\sum_{i=1}^{m}n_{i}d_{i}^{2}+3\left(3+1/n\right)\left(m+\sum_{i=1}^{m}d_{i}^{2}\right)\;,$$

which reduces to the identical

$$E\left(Z\right)\leq \left(3\xi^{3}\mathrm{lg}\xi \right)m^{2}+O\left(m\log m\right)$$

in case all the $n_{i}$’s and $d_{i}$’s are upper-bounded by some constant ξ, which again is on the order of $m^{2}$.

In addition to communication complexity, another natural efficiency measure in the random bit model concerns the *expected number of random bits* that needs to be drawn in order to achieve sampling. Randomness is needed in lines 3, 6 and 10 of Algorithm 4. A single random bit is required each time lines 6 and 10 are entered, but line 3 calls for sampling *X* according to distribution *q*. Let $V_{i}$ be the random variable that represents the number of random bits needed on the *i*th passage through line 3. For this purpose, we use the algorithm introduced by Donald Knuth and Andrew Chi-Chih Yao [21], which enables sampling within any finite discrete probability distribution in the random bit model by using an expectation of no more than two random bits in addition to the Shannon binary entropy of the distribution. Since each such sampling is independent from the others, it follows that $V_{i}$ is independently and identically distributed as a random variable *V* such that

(21) E(V)≤2+H(q)≤2+lgn,

where H(q) denotes the binary entropy of *q*, which is never more than the base-two logarithm of the number of atoms in the distribution, here *n*.

Let *R* be the random variable that represents the number of random bits drawn when running Algorithm 4. Reusing the notation of Section 2, let *S* be the random variable that represents the number of times variable *X* is sampled at line 3 and let $T_{i}$ be the random variable that represents the value of variable *T* upon termination of the *i*th instance of the **for** loop starting at line 9, for i∈{1,…,S}. The random variables $T_{i}$ are independently and identically distributed as the random variable *T* in Theorem 2 and the expected value of *S* is *C*. Since one new random bit is generated precisely each time variable *t* is increased by 1 in any pass through either **for** loops (line 5 or 9), we simply have

$$R=\sum_{i=1}^{S}\left(V_{i}+T_{i}\right)\;.$$

By virtue of Equations (3) and (21), Theorem 2, and using Wald’s identity again, we conclude that:

$$\begin{array}{cc} E(R) & =E\left(S\right)\left(E(V)+E(T)\right)\\ & \leq \left(1+2^{1-t_{0}}n\right)\left(\mathrm{lg}n+t_{0}+5+2^{-t_{0}}\right)\;. \end{array}$$

Taking $t_{0}=\left\lceil \;\mathrm{lg}n\right\rceil$ again, remote sampling can be completed using an expected number of random bit in O(lgn), with a hidden multiplicative constant no larger than 6. The hidden constant can be reduced arbitrarily close to 2 by taking $t_{0}=\left\lceil \;\mathrm{lg}n\right\rceil +a$ for an arbitrarily large constant *a*. Whenever target distribution *p* has close to full entropy, this is only twice the optimal number of random bits that would be required according to the Knuth–Yao lower bound [21] in the usual case when full knowledge of *p* is available in a central place rather than having to perform remote sampling. Note, however, that, if our primary consideration is to optimize communication for the classical simulation of entanglement, as in Section 3, choosing $t_{0}=\left\lceil \;\mathrm{lg}n\right\rceil -a$ would be a better idea because the summation in the left-hand term of Equation (13) dominates that of the right-hand term. For this inconsequential optimization, *a* does not have to be a constant, but it should not exceed lg(ξm), where ξ is our usual upper bound on the number of possible outcomes for each participant (if it exists), lest the right-hand term of Equation (13) overtake the left-hand term. Provided ξ exists, the expected number of random bits that needs to be drawn is linear in the number of participants.

The need for continuous random variables was not the only unrealistic assumption in Algorithms 1–3. We had also assumed implicitly that custodians know their private parameters precisely (and that the leader knows exactly each entry of density matrix ρ in Section 3). This could be reasonable in some situations, but it could also be that some of those parameters are transcendental numbers or the result of applying transcendental functions to other parameters, for example cosπ/8. More interestingly, it could be that the actual parameters are spoon-fed to the custodians by *examiners*, who want to test the custodians’ ability to respond appropriately to unpredictable inputs. However, all we need is for the custodians to be able to obtain their own parameters with arbitrary precision, so that they can provide that information to the leader upon request. For example, if a parameter is π/4 and the leader requests a *k*-bit approximation of that parameter, the custodian can compute some number $\hat{x}$ such that $|\hat{x}-\pi /4|\leq 2^{-k}$ and provide it to the leader. For communication efficiency purposes, it is best if $\hat{x}$ itself requires only *k* bits to be communicated, or perhaps one more (for the sign) in case the parameter is constrained to be between −1 and 1. It is even better if the custodian can supply a *k*-bit *truncation* because this enables the possibility to upgrade it to a (k+1)-bit truncation by the transmission of a single bit upon request from the leader, as we have done explicitly for the simulation of entanglement at line 19 of Algorithm 3 in Section 3.

Nevertheless, it may be impossible for the custodians to compute truncations of their own parameters in some cases, even when they can compute arbitrarily precise approximations. Consider for instance a situation in which one parameter held by a custodian is x=cosθ for some angle θ for which he can only obtain arbitrarily precise truncations. Unbeknownst to the custodian, θ=π/3 and therefore x=1/2. No matter how many bits the custodian obtains in the truncation of θ, however, he can never decide whether θ<π/3 or θ≥π/3. In the first case, x<1/2 and therefore the 1-bit truncation of *x* should be 0, whereas in the second (correct) case, x≥1/2 and therefore the 1-bit truncation of *x* is 1/2 (or 0.1 in binary). Thus, the custodian will be unable to respond if the leader asks him for a 1-bit truncation of *x*, no matter how much time he spends on the task. In this example, by contrast, the custodian can supply the leader with arbitrarily precise *approximations* of *x* from appropriate approximations of θ. Should a situation like this occur, for instance in the simulation of entanglement, there would be two solutions. The first one is for the custodian to transmit increasingly precise truncations of θ to the leader and let *him* compute the cosine on it. This approach is only valid if it is known at the outset that the custodian’s parameter will be of that form, which was essentially the solution taken in our earlier work on the simulation of the quantum *m*-partite GHZ distribution [1]. The more general solution is to modify the protocol and declare that custodians can send arbitrary approximations to the leader rather than truncations. The consequence on Algorithm 3 is that line 19 would become much more expensive since each custodian *i* would have to transmit a fresh one-bit-better approximation for the real and imaginary parts of the $d_{i}^{2}$ entries defining matrix $M_{iX_{i}}$. As a result, efficiency parameter γ(t) in Equation (6) would become

$$\gamma \left(t\right)=m+\left(t+2+\left\lceil 2\mathrm{lg}d\right\rceil +2\left\lceil \mathrm{lg}m\right\rceil \right)\sum_{i=1}^{m}d_{i}^{2}\;,$$

which should be compared with the much smaller (constant) value of γ given in Equation (12) when truncations of the parameters are available. Nevertheless, taking $t_{0}=\left\lceil \;\mathrm{lg}n\right\rceil$ again, this increase in γ(t) would make no significant difference in the total number of bits transmitted for the simulation of entanglement because it would increase only the right-hand term in Equations (14) and (20), but not enough to make it dominate the left-hand term. All counted, we still have an expected number of bits transmitted that is upper-bounded by $\left(3\xi^{3}\mathrm{lg}\xi \right)m^{2}+O\left(m\log m\right)$ whenever all the $n_{i}$’s and $d_{i}$’s are upper-bounded by some constant ξ, which again is on the order of $m^{2}$.

## 5. Discussion and Open Problems

We have introduced and studied the general problem of sampling a discrete probability distribution characterized by parameters that are scattered in remote locations. Our main goal was to minimize the amount of communication required to solve this conundrum. We considered both the unrealistic model in which arithmetic can be carried out with infinite precision and continuous random variables can be sampled exactly, and the more reasonable *random bit model* studied by Knuth and Yao [21], in which all arithmetic is carried out with finite precision and the only source of randomness comes from independent tosses of a fair coin. For a small increase in the amount of communication, we can fine-tune our technique to require only twice the number of random bits that would be provably required in the standard context in which all the parameters defining the probability distribution would be available in a single location, provided the entropy of the distribution is close to maximal.

When our framework is applied to the problem of simulating quantum entanglement with classical communication in its essentially most general form, we find that an expected number of $O(m^{2})$ bits of communication suffices when there are *m* participants and each one of them (in the simulated world) is given an arbitrary quantum system of bounded dimension and asked to perform an arbitrary generalized measurement (povm) with a bounded number of possible outcomes. This result generalizes and supersedes the best approach previously known in the context of multi-party entanglement, which was for the simulation of the *m*-partite GHZ state under projective measurements [1]. Our technique also applies without the boundedness condition on the dimension of individual systems and the number of possible outcomes per party, provided those parameters remain finite.

It would be preferable if we could eliminate the dependency of the expected number of bits of communication on the number of possible measurement outcomes. Is perfect simulation possible at all when that number is infinite, regardless of communication efficiency, a scenario in which our approach cannot be applied? In the bipartite case, Serge Massar, Dave Bacon, Nicolas Cerf, and Richard Cleve proved that classical communication can serve to simulate the effect of arbitrary measurements on maximally entangled states in a way that does not require any bounds on the number of possible outcomes [6]. More specifically, they showed that arbitrary povms on systems of *n* Bell states can be simulated with an expectation of $O(n2^{n})$ bits of communication. However, their approach exploits the equivalence of this problem with a variant known as *classical teleportation* [5], in which one party has full knowledge of the quantum state and the other has full knowledge of the measurement to be applied to that state. Unfortunately, the equivalence between those two problems breaks down in a multipartite scenario and there is no obvious way to extend the approach. We leave as an open question the possibility of a simulation protocol in which the expected amount of communication would only depend on the number of participants and the dimension of their simulated quantum systems.

Our work leaves several additional important questions open. Recall that our approach provides a bounded amount on the *expected* communication required to perform exact remote sampling. The most challenging open question is to determine if it is possible to achieve the same goal with a guaranteed bounded amount of communication *in the worst case*. If possible, this would certainly require the participants to share ahead of time the realization of random variables, possibly even continuous ones. Furthermore, a radically different approach would be needed since we had based ours on the von Neumann rejection algorithm, which has intrinsically no worst-case upper bound on its performance. This task may seem hopeless, but it has been shown to be possible for special cases of entanglement simulation in which the remote parameters are taken from a continuum of possibilities [3,8], despite earlier “proofs” that it is impossible [2].

A much easier task would be to consider other communication models, in which communication is no longer restricted to being between a single leader and various custodians. Would there be an advantage in communicating through the edges of a complete graph? Obviously, the maximum possible savings in terms of communication would be a factor of 2 since any time one participant wants to send a bit to some other participant, he can do so via the leader. However, if we care not only about the total number of bits communicated, but also the *time* it takes to complete the protocol in a realistic model in which each party is limited to sending and receiving a fixed number of bits at any given time step, parallelizing communication could become valuable. We had already shown in Ref. [1] that a parallel model of communication can dramatically improve the time needed to sample the *m*-partite GHZ distribution. Can this approach be generalized to arbitrary remote sampling settings?

Finally, we would like to see applications for remote sampling outside the realm of quantum information science.

## Abbreviations

The following abbreviations are used in this manuscript:

i.i.d.	independent identically distributed
GHZ	Greenberger–Horne–Zeilinger
POVM	positive-operator valued measure
